# Asthma and Allergy: Unravelling a Tangled Relationship with a Focus on New Biomarkers and Treatment

**DOI:** 10.3390/ijms23073881

**Published:** 2022-03-31

**Authors:** Pablo Rodriguez del Rio, Andrew H. Liu, Magnus P. Borres, Eva Södergren, Fabio Iachetti, Thomas B. Casale

**Affiliations:** 1 Department of Allergy, University Children’s Hospital Nino Jesús, 28040 Madrid, Spain; 2 Breathing Institute, Section of Pediatric Pulmonary & Sleep Medicine, Children’s Hospital Colorado, Aurora, CO 80045, USA; andrew.liu@childrenscolorado.org; 3 Department of Pediatrics, University of Colorado School of Medicine, Aurora, CO 80045, USA; 4 Department of Pediatrics, National Jewish Health, Denver, CO 80217, USA; 5 Thermo Fisher Scientific, 75123 Uppsala, Sweden; magnus.borres@thermofisher.com (M.P.B.); eva.sodergren@thermofisher.com (E.S.); fabio.iachetti@thermofisher.com (F.I.); 6 Department of Women’s and Children’s Health, Uppsala University, 75123 Uppsala, Sweden; 7 Morsani College of Medicine, University of South Florida, Tampa, FL 33612, USA; tbscasale@usf.edu

**Keywords:** asthma, allergy, molecular diagnosis, biomarkers, EDN, treatment

## Abstract

Asthma is a major driver of health care costs across ages. Despite widely disseminated asthma-treatment guidelines and a growing variety of effective therapeutic options, most patients still experience symptoms and/or refractoriness to standard of care treatments. As a result, most patients undergo a further intensification of therapy to optimize symptom control with a subsequent increased risk of side effects. Raising awareness about the relevance of evaluating aeroallergen sensitizations in asthmatic patients is a key step in better informing clinical practice while new molecular tools, such as the component resolved diagnosis, may be of help in refining the relationship between sensitization and therapeutic recommendations. In addition, patient care should benefit from reliable, easy-to-measure and clinically accessible biomarkers that are able to predict outcome and disease monitoring. To attain a personalized asthma management and to guide adequate treatment decisions, it is of paramount importance to expand clinicians’ knowledge about the tangled relationship between asthma and allergy from a molecular perspective. Our review explores the relevance of allergen testing along the asthma patient’s journey, with a special focus on recurrent wheezing children. Here, we also discuss the unresolved issues regarding currently available biomarkers and summarize the evidence supporting the eosinophil-derived neurotoxin as promising biomarker.

## 1. Introduction

Asthma is a major driver of health care costs for all ages and the most common chronic disease for children and adolescents [1,2]. The prevalence of asthma has risen steadily over the past decades, currently reaching about 300 million people worldwide and potentially involving a further 100 million by 2025 [3,4].

Despite the availability of both widely disseminated asthma-treatment guidelines and an ever-evolving variety of effective therapeutic options, most patients with asthma continue to experience symptoms, with one in two adults reporting very poorly controlled asthma [5]. Similarly, the prevalence of inadequately controlled asthma in the pediatric population is quite high, with 30–40% of all severe exacerbations occurring in children and adolescents. [6]. It has been well documented that a significant proportion of patients might be refractory to standard of care treatments, resulting in further intensification of therapy to optimize symptom control, thus remaining symptomatic despite maximal therapy and subsequent increased risk of side effects [7].

To date, implementation of existing guidelines has been inadequate, particularly in primary care setting [8], and due to several barriers, there is limited emphasis on identifying triggers or allergens, jeopardizing efforts to effectively improve asthma control and patients’ burden [9]. Such a management approach seems to not fully acknowledge that the allergies are frequent triggers of asthma exacerbations [10] and up to 80% of childhood asthma and more than 50% of adult asthma cases may have an allergic component [11]. To date, in the USA, allergy evaluation has been discussed in about 33% of primary care office visits for asthma with allergy testing being only documented in 2% of cases of asthma over the course of a year [12].

Considering a precision medicine approach, to improve asthma patient care is paramount to bridge the observable characteristics (phenotypes) with the mechanisms driving the disease (endotypes) using biomarkers. While an endotype-driven treatment still needs to face multiple challenges before its implementation in daily clinical practice [13], asthma endotype classifications combined with specific biomarkers may hold great potential for new therapeutic modalities and better treatment efficacy [14]. Although specific, sensitive, and reliable (point-of-care) biomarkers would be critical for selecting the proper treatment for a given patient, current biomarkers appear to be good indicators of T2 endotypes but not strong predictors of response to targeted treatments [15] and most of them require further validation [16].

In this narrative review, we explore the tangled relationship between asthma and allergy from a molecular perspective, and the relevance of allergen sensitization and testing in asthma diagnosis and therapy selection, with a special focus on recurrent wheezing children. Here, we also discuss the unresolved issues regarding current biomarkers in clinical practice and summarize the evidence supporting eosinophil-derived neurotoxin (EDN) as promising biomarker.

## 2. Pursuing an Optimized Asthma Care: The Relevance of the Molecular Approach

Asthma is recognized as a disease with significant heterogeneity in clinical features (phenotypes), disease severity, pattern of underlying disease mechanisms and responsiveness to specific treatments (e.g., responder/non-responder, corticosteroid sensitive or resistant). Thus, precision medicine strategies are needed to better tailor therapy on a patient’s clinical and immunological profile [17]. Accordingly, classifying asthma into distinct endotypes in a laboratory- and clinical-evidence-based manner contributes to personalized precision medicine by unravelling disease mechanisms. To date, asthma has been classically associated with type 2 inflammation, characterized by high levels of immunoglobin E (IgE), eosinophils, fractional exhaled nitric oxide (FeNO), and cytokines frequently found in allergic responses including interleukin 4, 5, 13 and 9 (IL-4, IL-5, IL-13, IL-9). However, none of these biomarkers have proven effective in differentiating responses between specific drugs that target type 2 inflammation. Moreover, between 10 and 33% of subjects with asthma are not associated with allergy (non-allergic asthma) and exhibit non-type 2 inflammation (non-T2 or T2-low endotype) with a prevalence of neutrophils or a paucigranulocytic pattern. Therefore, there remains an unmet clinical need in the study of the mechanisms and biomarkers for both T2-high and T2-low endotypes as concerns their ability to predict response to targeted therapy [18].

It has been recently proposed that the molecular allergology approach to allergic asthma may contribute to a better understanding of disease mechanisms, a precise diagnosis through the description of the molecular allergen sensitization profile, as well as to an optimal selection of responders to the targeted treatment, either with allergen immunotherapy (AIT), or with biologicals [19]. Importantly, it has been recently recognized that the heterogeneity of asthma can be mostly ascribed to the complex interactions between the host and the environment, including aeroallergens [20]. To this end, defining the allergen sensitization of a patient with asthma at the molecular level by measuring specific IgE to purified natural or recombinant allergens can improve diagnostic accuracy and improve asthma phenotyping [21]. Allergen components have been available for testing in the clinic for almost two decades. The different allergenic proteins in some pollens and perennial allergens (e.g., dust mite) were characterized early. Pet-derived allergens are the third leading cause of respiratory allergies, after mites and pollens, and a significant number of new findings are changing the understanding of this allergy. Of note, the prevalence of sensitization to dander from various animals appears to be increasing worldwide, with 1 in 5 adults being sensitized to cats. More recently, the allergenic proteins for cats and dogs have been characterized with three important lipocalins (Canis familiaris 4, 6 and Felis domesticus 7) being made available for testing as late as last year [22,23].

Furthermore, molecular-based diagnostics has a direct effect on the strategy of choosing which allergens should be used in AIT [24]. Of note, it is recognized that molecular diagnosis allows for a personalized AIT thus sparing patients the burden of multiple treatments or unnecessary lifestyle modifications involved in allergen avoidance [25]. In this scenario, the component resolved diagnosis (CRD) stands as a promising starting point as it allows to precisely identify the number and type of recognized molecules in the individual patient that are clinically relevant.

Among the innovative molecular approaches to allergic asthma, epigenetics is gaining significant interest by virtue of its role in immune cell differentiation and plasticity [26] and given the observation that airway epithelium pathobiology in asthma is regulated by epigenetic mechanisms [27]. Importantly, epigenetic modifications can mediate the effects of the environment on the development of or protection from allergic diseases [26]. Accordingly, DNA methylation changes might thus be used as molecular biomarkers to quantify the different allergy enhancing or protective exposures [28]. Epigenetic mechanisms, including methylation, can also contribute to childhood asthma; therefore, identifying DNA methylation profiles in asthmatic patients can inform disease pathogenesis. Of note, recent analyses from the MeDALL (European Mechanisms of the Development of Allergy) consortium reported a significant association between reduced whole blood DNA methylation at both 21 and 14 CpG sites and childhood allergy, thus providing novel insights into the shared molecular mechanisms underlying asthma, rhinitis, and eczema [29,30].

## 3. The Relevance of Allergen Testing in Asthma Management

### 3.1. Diagnosis

It is increasingly recognized that evaluating aeroallergen sensitization in asthma patients is a key step to improve patients’ care [31]. In line with this, the National Institute of Health Asthma Outcomes Task Force recommends the assessment of aeroallergen sensitization as a core biomarker for classification of asthma [32] while recommendations from the GA^2^LEN/EAACI state that the clinical relevance of each allergen is more important than the number of sensitizations itself [33]. Although the testing criteria and the timing of the testing (either alongside or after a diagnosis of asthma has been made) may vary among guidelines, integrating aeroallergen evaluation into asthma management is of paramount importance to optimize the asthma patient journey from diagnosis to treatment.

Different types of aeroallergens and specific sensitization profiles are associated with a different pattern of clinical symptoms and different levels of severity. It has been documented that there is a direct relationship between the degree of allergen sensitization, measured as serum specific IgE, and the likelihood of expression of asthma symptoms [34,35]. This association with allergen-specific IgE titers is especially marked in children, where elevated specific IgE supports a T2-high profile. Of note, when associated with childhood symptoms of atopy and asthma, positive IgE testing aids in diagnosing early-onset allergic asthma [36].

Testing for molecular aeroallergen sensitization can be of help to identify individuals who are sensitized to minor allergens or to cross-reactive allergens as well as to confirm two or more coexisting sensitizations (polysensitization). Most patients with allergic asthma seen by specialists are polysensitized, and many of them are also poly-allergic (sensitizations with clinical relevance) because polysensitization does not necessarily mean that all sensitivities are clinically significant. To date, while in Europe allergen immunotherapy practice recommends that 1 or 2 of the most important allergens for the patient are to be included in the same extract, in the United States, mixtures including many or most of the sensitizing allergens are commonly administered [25].

Aeroallergen sensitization testing enhances the ability to predict asthma development, drug response, and risk for future asthma exacerbations in children. In addition, among children and adolescents, the allergy testing may help identify common comorbid conditions such as allergic rhinitis, which when treated appropriately, can improve asthma control [37]. Within the pediatric population, preschool children with repeated wheeze have been recently included among the patients’ profiles who may benefit the most from aeroallergen sensitization evaluation [31]. As recently proposed by Casale et al., several patients’ profiles should undergo aeroallergen testing: persistent asthmatics, patients who need oral corticosteroids (OCS) or inhaled corticosteroids (ICS), patients who seek to get advice on the presence of pet at home or regular pet contact or to better understand their condition, and patients who may be eligible to receive AIT or biologicals [31].

Several allergen component tests are now available for clinicians to use in everyday practice [38]. Allergen-specific IgE tests utilizing individual allergenic molecules are regarded as a more precise and informative option, particularly in polysensitized patients compared to those tests based on whole allergenic extracts. A growing spectrum of molecules, representing single allergens of clinical relevance, have been identified, characterized, and produced for commercial in vitro assays. CRD can be of special help in unveiling co-sensitization and/or cross-sensitization of closely related or widely different allergen sources [39,40]. Furthermore, CRD can be of special interest when the prescription of AIT needs to be accomplished in areas with high frequency of sensitization to “minor allergens” [41]. In addition, this is relevant when one should identify whether the sensitization is primary (species-specific) or a result of cross-reactivity to proteins with similar protein structures. In this regard, CRD is gaining greater recognition for pet allergy diagnostics [42]. To date, the frequency of co-sensitization with cat and dog may be explained by shared proteins between the two species e.g., lipocalins, or serum albumins (Table 1).

Four dog allergens (e.g., Can f 1, Can f 2, Can f 4, and Can f 6) and two cat allergens (Fel d 4 and Fel d 7) are in the lipocalins family of proteins [42]. A recent study carried out in 294 children and adults with suspected asthma and a positive skin prick test to cat and dog showed that allergen components can unveil the molecular basis of animal polysensitization and may be of help in both identifying primary sensitizers and explaining how individual IgE patterns of expression may correlate with previous pet ownership [46].

### 3.2. Therapy Selection

Asthma management entails allergen avoidance: therefore, identifying allergen sensitization is crucial to both foster education on allergen avoidance and guide appropriate exposure control [47]. Patients with asthma undergoing allergy testing are more likely to adopt preventive measures (including an asthma plan, trigger avoidance, and medication adherence), thus experiencing fewer days with allergy symptoms than their counterparts who had not been tested [48]. These outcomes were supported by a study of adults with moderately severe asthma, who had an individualized plan, including environmental control based on the results of allergy testing [49]. Although avoidance interventions at the population level for the main allergens are not supported by robust evidence of efficacy and are still somewhat controversial, knowing which specific allergen is triggering the allergic asthma can be of help in preventing the appearance or worsening of bronchial symptoms in sensitized selected individuals [50]. Importantly, it is key not only to employ appropriate avoidance measures, but also to effectively sustain these interventions over time to attain a long-lasting symptom improvement.

As suggested by Global Initiative for Asthma (GINA) guidelines, most asthma patients react to multiple triggers that are ubiquitous in the environment, thus making their avoidance very burdensome for the patient. Inhalant allergens, including indoor (molds, animal dander, and house dust mites) and outdoor ones such as pollens, appear to be the most important for children and adults with asthma. However, medications to maintain good asthma control have a key role because patients appear less affected by environmental factors when their asthma is well controlled [10].

Allergen testing can predict response to corticosteroids in both children and adults. Certain patterns of inhaled allergen sensitization in early life may help identify children at high risk for severe exacerbations and those who are likely to respond well to ICS [51]. Similarly, a recent study in the adult population with asthma and sensitization to airborne allergens found that among sensitized subjects, the decline in forced expiratory volume in 1 second (FEV_1_) was lower for long-term ICS users (1.3–8 and >8 years), compared with nonusers and short-term users [52].

Identifying sensitizations in allergic rhinitis subjects may also help guide AIT for potential asthma prevention. Therefore, positive specific IgE is considered a useful biomarker for AIT candidate selection in the context of a clear history of symptoms on exposure to the relevant allergen [25]. To date, AIT stands as the only treatment approach able to alter the natural course of allergic respiratory diseases by decreasing frequency and severity of symptoms and progression of rhinitis to asthma [25,53]. As AIT works through induction of allergen-specific tolerance, its effectiveness relies on the inclusion of relevant allergens in adequate concentrations to achieve tolerance. As a result, omission of relevant allergens or the use of irrelevant allergens may reduce efficacy of immunotherapy.

Polysensitization develops over time and has clinical relevance for treatment decisions. In many patients originally classified as polysensitized based on SPTs to whole extracts, molecular diagnosis could identify clear sensitizations to major allergens with clinical relevance to be considered for AIT [54]. A detailed molecular diagnosis will add value when determining whether AIT is appropriate for a given patient and, if so, which allergen(s) should be administered. A study performed on 141 patients with pollen-induced AR [55] showed that molecular diagnosis would change the allergen composition in AIT. The patients were tested with SPTs and the ISAC^®^ microarray-based panel of allergens, and prescriptions before and after the knowledge gained with the input of ISAC were compared. In 1 out of 2 cases, the allergen composition of AIT was changed due to the molecular diagnosis results.

Finally, one must bear in mind that that even negative results for aerollergen sensitization can be meaningful to personalize asthma care, as patients will be spared from taking drugs that will not be effective and from the inconvenience of avoiding triggers to which they are not sensitized [31]. Last but not the least, negative test results may prompt a search for other causes of the observed symptoms, thereby improving asthma phenotype definition and a personalized therapeutic approach.

## 4. Biomarker Hunting: From Current Challenges to Promising Candidates

Asthma had been tackled for a long time with a “one size fits all” management, although it encompasses a wide range of phenotypes differing in severity and natural history. Therefore, elucidation of these phenotypes and identification of biomarkers with which to recognize them and guide appropriate treatment remain a priority. Biomarkers stand as measurable indicators, bridging an underlying pathway to a phenotype or endotype of a disease, and are of great value to both predict treatment response and monitor disease progression. Therefore, an ideal biomarker should be sensitive, specific, able to provide positive and negative predictive values, while being simple to measure and cost-effective.

Currently available biomarkers allow one to dichotomize individuals to either T2-high or non-T2-high groups, thus establishing criteria for therapy selection in routine practice. Of note, some biomarkers may offer highest yields when applied in the background of certain clinical characteristics (e.g., more reversible airway disease, age at disease onset). Therefore, a combination of different biomarkers, or a biomarker panel, may be more suitable than one to refine selection of biological therapies. Exhaled NO, blood eosinophils, serum IgE, and periostin are well-studied and established biomarkers for T2-high asthma endotype [36].

FeNO measurement can be a valuable tool as it predominantly signifies IL-4 and IL-13 activity, with FeNO concentrations greater than 50 ppb (>35 ppb in children) unveiling an eosinophilic airway inflammation and ICS responsiveness [56]. In conjunction with peripheral eosinophilia, an elevated FeNO is a risk factor for airway hyperreactivity and uncontrolled asthma [57]. However, despite being a reasonable indicator of T2-driven asthma, there are no specific guidelines on how FeNO can guide therapy with biologics and FeNo may be affected by relevant confounders including smoking, dietary nitrate intake, and virus infections. Nevertheless, a recent post hoc analysis of the Liberty Asthma Quest study evaluated the additional value of baseline FeNO, adjusted for baseline eosinophil level and other clinical characteristics, as a predictor of response in dupilumab-treated patients with uncontrolled, moderate-to-severe asthma [58].

By virtue of IgE’s role as a key mediator in the development and maintenance of allergic inflammation and of a documented association between allergic asthma and elevated total IgE [11], serum total IgE has been proposed as a predisposing factor for allergic asthma and often employed for epidemiological analyses. However, elevated levels of serum total IgE are not only found in patients with allergic asthma but also with other conditions including parasitic infestations, inflammatory diseases, and some primary immunodeficiency diseases [59]. Accordingly, total levels of IgE should be carefully interpreted and not considered as an indication for the presence of allergic diseases. In terms of diagnostic and therapeutic relevance, a recent study proposed serum-free IgE rather than total IgE as biomarker of type 2 asthma in adults [60] and suggested that it could be used to determine therapeutic response. Given the emerging availability of biologicals targeting type 2 cytokines, it is important to assess total IgE ability to assist clinicians in choosing a biological therapy among the approved options. As suggested by Sanchez et al., the available evidence indicates that total IgE, as well as the blood eosinophil count and FeNO, are not enough to select one therapy over another [61]. The presence of specific IgE antibodies to environmental allergens proves sensitization and is associated with allergic asthma. However, it has been suggested that the interpretation of skin prick tests and specific IgE to whole allergen extracts relies on arbitrary cutoffs, which do not distinguish between clinically relevant and non-relevant sensitizations [11]. In addition, while positive serum specific IgE levels and symptoms upon exposure to the sensitizing allergen are indicated as inclusion criteria for starting AIT, specific IgE levels solely were found a poor predictor of AIT indication. Overall, the clinical relevance of total serum IgE and specific serum IgE as biomarker for therapy selection requires further studies.

Although its expression in bronchial tissue is not associated with asthma severity, periostin has been shown to be a biomarker of persistent eosinophilic airway inflammation despite corticosteroid use [62]. The potential use of serum periostin levels is the assessment of greater response to anti-T2-based therapies. Of note, periostin disadvantages encompass the presence of several periostin splice variants, that complicate its detection, and the uncertainty regarding its use as potential biomarker in children, since baseline periostin levels are higher in children given the ongoing bone growth during childhood.

The measurement of eosinophilia in peripheral blood cells (either by absolute count (BEC) or by percent differential of total leukocytes) has been investigated as a potential surrogate marker of bronchial and/or lung inflammation. Higher eosinophil counts identify patients with more severe disease and poorer outcomes, patients for whom biologic therapies targeting allergic and/or eosinophilic pathways are recommended. To date, the recent European Academy of Allergy and Clinical Immunology (EAACI) guideline on the use of biologicals in severe asthma suggested that the higher the blood eosinophils, the higher the expected impact of benralizumab, dupilumab, and mepolizumab in reducing severe asthma exacerbations as well as the higher improvement in asthma control in patients treated with benralizumab and reslizumab. In contrast, the effect of omalizumab on exacerbations did not depend on blood eosinophils [63,64,65], although a greater response in patients treated with omalizumab, when peripheral blood eosinophils were ≥300 cells·μL^−1^, was also reported [66].

Although blood eosinophil numbers can easily and quantitatively be determined in any hospital laboratory, standardization of the appropriate cut-off of clinically relevant eosinophilia and the need for single or multiple measurements in different settings are needed prior to the application in clinical practice. In addition, some confounding factors should be acknowledged for blood eosinophils, including circadian variation, parasites (e.g., helminthiases, schistosomiasis, filariases), and treatment with systemic corticosteroids [15]. For example, a diurnal variability of BECs has been reported, with peak counts recorded soon after midnight and lowest counts at noon. Regarding the blood eosinophil counts variability, a recent clinical trial conducted in patients with severe uncontrolled asthma receiving standard maintenance treatment showed that most patients shifted to a different BEC group from their baseline group at some point during the study and such behavior was most marked in patients receiving long-term OCS. Therefore, the need for several measurements of BEC may be particularly relevant for patients receiving OCSs [67,68,69].

Biomarkers’ clinical value also relies on their ability to both predict treatment response and monitor disease progression. While circulating blood eosinophil count of ≥300/μL may be helpful to identify a T2 immune biology to initiate therapy with an anti-T2 mAb, it has very limited value to monitor response [70,71]. Overall, peripheral eosinophil count is commonly obtained due to ease of performance and clinical accessibility. However, one should keep in mind the caveats in its use and the potential limitations as a biomarker for phenotyping refractory asthma or selecting therapy in mild asthma.

Overall, more differentiating, noninvasive, simply measurable, reliable biomarkers with well-defined cutoff values and documentation on their stability/behavior over time are urgently needed. Research focusing on eosinophil-derived molecular products is now emerging, which highlights more potential biomarkers for allergic diseases [15].

Eosinophil granular proteins are a useful eosinophilic activation marker in asthmatic patients and include the eosinophil peroxidase (EPO), eosinophil cationic protein (ECP) and EDN [72]. Earlier studies reported that in patients with asthma serum levels of EPO negatively correlated with FEV_1_ and positively with the number of eosinophils in peripheral blood [73]. Of note, it can be measured in saliva and is documented correlating with sputum eosinophils. In addition, in asthmatic children, EPO levels were lower compared to healthy controls and positively correlated with total IgE levels [74].

ECP has been proposed as a marker for eosinophilic disease and quantified in biological fluids including serum, bronchoalveolar lavage, and nasal secretions. Elevated ECP levels are found in allergic asthma [75] and, among asthmatics, were associated with high neutrophil count [76]. ECP levels are affected by age, smoking, circadian rhythm, and seasonal variation, although only smoking appears to be of clinical significance [77]. Serum ECP was significantly higher in children with symptomatic asthma than in asymptomatic patients [78]. In children, ECP may provide complementary value when used together with lung function test and FeNO. A recent study suggested that plasma ECP concentrations may be a useful marker of type 2 inflammation in children and may help identify those children at highest risk for recurrent exacerbations who could benefit from corticosteroid treatment [79].

EDN, also known as eosinophil protein X, is a granular protein released from activated eosinophils [72]. Patients with severe asthma and uncontrolled asthmatics exhibit higher serum EDN level which showed a good positive correlation to total eosinophil count (TEC) [80], thus suggesting the use of EDN as biomarker of asthma severity in adults. A recent study carried out in adults from the Epidemiological Study on the Genetics and Environment of Asthma revealed that EDN could be a potential biomarker to monitor asthma evolution in adults as its levels are associated with different asthma expression patterns in adults [71]. It has been also reported that higher serum EDN levels could be found in children at the acute phase than at the stable phase of asthma, and in contrast to TEC, the serum EDN level can be predictive of the severity of asthma [81]. In the pediatric population, EDN may hold great promise in distinguishing persistent wheezing children from children presenting wheezing triggered by respiratory tract infections [82] and in aiding in the diagnosis of school age childhood asthma [83] as well as to monitor response to montelukast or budesonide in preschool children with asthma [84]. Given the limited ability of BEC to monitor treatment response to biologicals, the observed significant correlation between reduced serum EDN level from baseline and lung function improvement after omalizumab, benralizumab and reslizumab [85,86,87] may help monitoring the treatment response to IgE- and IL-5 targeted therapies.

Finally, from a clinical standpoint, the finding that EDN levels can be measured in multiple specimen types, such as sputum, serum, urine, bronchoalveolar lavage fluid, and nasal lavage fluid [88,89,90,91,92] and, in contrast to BEC, are less affected by circadian rhythm or gender differences [93] expand its potential usefulness in routine practice. Table 2 illustrates the relative pros and cos of available Type 2 asthma biomarkers.

## 5. The Wheezing Child: A Paradigm to Optimize Asthma Control in Adult Life

The global prevalence of current wheeze in children and adolescents was estimated to be 14.1% and 11.7% and projected to increase by 0.06% and 0.13% annually, respectively [95,96]. Wheezing is common among preschool children and infants; of note, young children with recurrent wheezing encompass a heterogeneous group with different genotypes and phenotypes that lead to different outcomes [97]. Overall, recurrent wheezing is associated with a two-fold increase in outpatient physician consultation or even the emergency department, an up to 5-fold greater risk to be admitted to hospital [98], missed days at school, activity limitation, and sleep disturbances [99]. Several phenotypes of preschool wheeze have been proposed to identify individuals at risk for persistent asthma at school age and display a temporal pattern of symptoms (Table 3).

Using the latent class analysis (LCA) technique, Fitzpatrick et al. identified four phenotypes of recurrent wheezing in preschool children based on type-2 inflammatory features including BEC, atopic eczema, aeroallergen, and food sensitization and/or pet exposures [51]. These phenotypes are distinguishable with regards to exacerbation risk, with inhaled allergen sensitization patterns being important risk factors, and, importantly, predict favorable response to daily ICS treatment to prevent exacerbations. Thus, certain patterns of inhaled allergen sensitization in early life can help identify children at high risk for severe exacerbations and those who are likely to respond well to ICS [51]. Of note, ICS are recommended as first choice of controller treatment in all preschool recurrent wheezing children irrespective of phenotype, but they are particularly beneficial in terms of fewer exacerbations in atopic children [106].

Genes and environmental factors such as respiratory viruses, tobacco smoke exposure, and inhaled allergens can modify the phenotypes of early childhood wheezing [107]. Furthermore, early exposures, including that to older siblings, pets, farm animals, and house-dust endotoxin, seem to influence the risk for persistent asthma [108,109]. To date, children suffering from allergic asthma, especially those with a persistent moderate and severe phenotype, were more often sensitized to all the three major dust mite allergens (Der p1, Der p2, Der p23) [110]. Of note, Der p 23 sensitization has been recently described as being associated with increased asthma risk [111]. Although many individuals later diagnosed with asthma exhibit their first symptoms during the preschool period, diagnosing asthma in preschool children is challenging, resulting in undertreatment of young asthmatic children and possibly overtreatment of transient wheezers [112]. Of note, transient early wheezing and nonallergic wheezing generally subside by 4–6 years of age as the airways enlarge in a growing child [31]. It is well documented that a lack of diagnostic criteria as well as the incapability of pre-school age children to perform conventional lung function tests, like exhaled nitric oxide or spirometry, hinder effective diagnosis and assessment in children [113,114]. As diagnosing asthma in the pediatric population is still challenging in clinical practice, defining the prognosis of preschool children requiring medical attention for recurrent wheezing appears of great value to optimize asthma care.

The progression from recurrent wheezing to persistent asthma is variably predicted by factors that emerged in cohort studies including the early sensitization to inhalant or food allergens, atopy, family history of asthma male gender, peripheral blood eosinophilia, as well as a history of wheezing with lower respiratory tract infections. The Asthma Predictive Index (API), that includes recurrent wheeze (e.g., more than three wheezing episodes per year) with risk factors such as parental asthma, atopic dermatitis, and allergen sensitization in the child, may more effectively identify young persistent asthma risk [115]. Overall, API has an appreciable likelihood ratio (~7.4) as it increases the probability of a prediction of asthma by 2–7 times [106,116]. Children with a positive API were found 7 times more likely to have active asthma at school age [117]. A modified version of API, modified API (mAPI), allows clinicians to identify children from birth to age 4 at high risk of developing asthma as well as to predict children response to ICS [118,119]. Importantly, mAPI acknowledges the contribution of allergic sensitization to at least one aeroallergen as a major criterion.

The identification of sensitization patterns based on individual allergens and linking these to disease morbidity for asthma in young children could have relevant clinical implications. Data from German MAS birth cohort study showed that schoolchildren sensitized to perennial allergens with high exposure early in life are more prone to develop an impaired lung function at school age than children without sensitization or sensitized to indoor allergens but with a low exposure [120]. Therefore, diagnosing specific inhalant allergen sensitizations in at-risk children can aid in designing allergen-avoidance strategies [121] as well as in guiding exposure mitigation and AIT choice in asthma prevention if allergic rhinitis is present. Allergen sensitization also negatively affected asthma outcomes in children with poorly controlled asthma and sensitization as they were more likely to use oral corticosteroids than their counterparts without allergen sensitization [122].

Furry animals, such as dogs and cats, represent important allergen sources [42]. It has been reported that sensitization to dog, cat, and horse throughout childhood was significantly associated with asthma at age 7 years [123]. In a population-based study with 259-animal sensitized, most were sensitized to two or more dog allergens, and co-sensitization to Can f 5 [prostate (i.e., male) specific allergen] and Can f 1/2 conferred the greatest risk for asthma [124]. In line with this, a recent study in children with mild to severe asthma showed that sensitization to a greater number of dog components may identify children whose asthma severity is driven by dog exposure and may benefit from targeted interventions such as exposure mitigation and immunotherapy [125]. To this end, molecular-based allergy diagnostics such as CRD provides clinicians with opportunities to better characterize children with polysensitization [126]. Nevertheless, one should keep in mind that a disagreement currently exists between asthma guidelines on the routine use of allergy testing in the diagnostic work-up of a child with persistent asthma [127]. Collectively, healthcare providers dealing with a preschool child with recurrent wheeze may rely on several tools, such as mAPI, aeroallergen testing, and CRD, to effectively predict the risk of persistent asthma in later childhood and adulthood. Armed with this predictive information, personalized education and successful implementation of allergen avoidance and appropriate therapies can help to reduce the substantial burden associated with more severe, persistent, and exacerbation-prone disease. From a clinical standpoint, a recurrent wheezing child may be a useful paradigm to optimize asthma care in children at high risk of developing persistent, life-long asthma.

## 6. Conclusions

**Figure 1:**
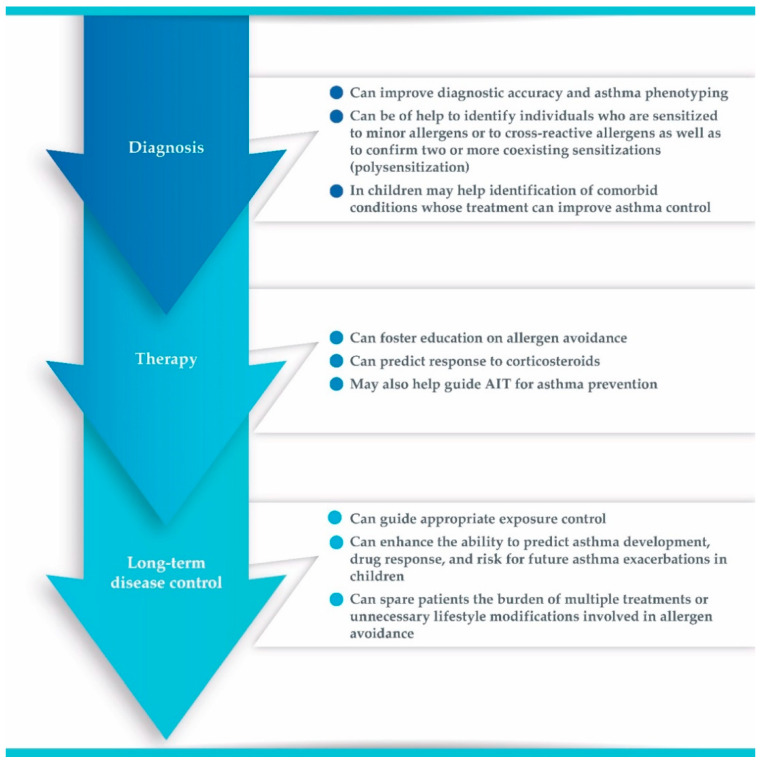
Relevance of allergen testing across asthma patient journey. AIT, allergen immunotherapy.

Asthma affects both children and adults and displays high morbidity. As asthma should be regarded as a syndrome, because of its phenotypic and endotypic heterogeneity, therapeutic approaches can be most effective if tailored to selected molecular targets in the dysregulated pathways of that given patient [128]. Despite growing therapeutic armamentarium, asthma control remains largely inadequate, and rates of healthcare use are high in both pediatric and adult populations, thus posing a substantial burden on societies. It has been suggested that raising awareness about the relevance of evaluating aeroallergen sensitizations in asthmatic patients is a key step in improving asthma/allergy care and informing clinical practice [31]. To date, mounting evidence is supporting the relevance of aeroallergen testing across the asthma patient journey (Figure 1).

Nevertheless, allergic sensitization should be regarded as a quantifiable rather than dichotomous trait, e.g., we can use the titer of allergen-specific sIgE antibodies and/or the number of sensitizations to the major allergen components of an exposure (e.g., dog) [129]. Physicians are currently provided with tools to better describe sensitization such as CRD that measures sIgE response to many major and minor allergens. This approach aids in refining the relationship between sensitization, clinical outcomes, and asthma progression [130]. CRD stands as a useful tool to better characterize children with polysensitization, to offer guidance in pet allergy treatment recommendations, including exposure remediation and AIT prescription [42,126] based on major components present in allergenic extracts used in AIT and the patient’s profile. The information retrieved through molecular allergy diagnostics opens novel avenues to advance asthma care while raising concerns about the correct timing for its application [131]. To implement the CRD approach in the diagnostic and management algorithms for asthma, appropriate interpretation tools should be developed to facilitate its use [129].

To unravel asthma molecular networks and to link phenotype with endotypes, great efforts have been placed in the search of biomarkers endowed with several features such as ease of performance, reliability, clinical accessibility, and the ability to predict outcome and disease monitoring [15]. Despite drawbacks of currently employed biomarkers, therapy selection still relies on them, as also documented in international guidelines and opinion papers [14,40,53,63,64,65]. Total IgE, FeNO, and BEC are the main drivers for biologicals prescription in patients with moderate to severe T2 high asthma with established cut off values for likelihood of treatment response.

Furthermore, we are still lacking more effective biomarkers for therapy selection in patients with overlapping phenotypes or in those for whom the presence of risk factors, such as smoking, or of concomitant therapies (e.g., systemic corticosteroids), which strongly hinders the reliability of some biomarker assessments. In this challenging scenario, EDN is an analytically attractive biomarker that can be reliably quantified in multiple specimens [93] and is stable during long-term storage. Of note, EDN may hold promise in distinguishing wheezing children from children with respiratory tract infections [82] and in aiding in the diagnosis of school age childhood asthma [83].

Future studies addressing the prognosis of preschool recurrent wheezing children appear of great value to optimize asthma care. It has been well established that diagnosis asthma in preschool children remains an unsolved issue. To date, preschool children with recurrent wheezing display several phenotypes (reflecting different endotypes and differing for clinical manifestations, natural history, response to therapy and inflammatory mechanisms) and may also switch between phenotypes or progress into another. Furthermore, not all the observed phenotypes are precursors of or manifestations of childhood asthma and, when it is the case, may be differentially responsive to asthma-targeted therapies. To this end, it has been advised to distinguish children based on allergen sensitization, evidence of eosinophilia and evidence of neutrophilia and/or infection [98]. In line with this, efforts should also be directed towards a better characterization of children with polysensitization and a routine implementation of allergen testing in at-risk children to design allergen-avoidance strategies as well as to guide AIT choice in asthma prevention. Another area deserving further investigation is the search of biomarkers for nonallergic type2-low children as the type-2 low inflammatory endotype is still poorly characterized in the pediatric population. Finally, it is important to recognize that asthma phenotypes are not static and can change with time in individual patients. Therefore, continued assessment of patients unresponsive to therapies requires reassessment of relevant biomarkers.

## Figures and Tables

**Table 1:** Major and minor dog and cat allergens. Can f, canis familiaris; Equ c, equus caballus; Fel d, felis domesticus; IgE, immunoglobin E. Elaborated from data in [37,42,43,44,45].

Molecular Allergen	Allergen Family	Biological Function	Sensitization Rate (%) *	Cross-Reactivity	Sensitizer Role
DOG, CANIS FAMILIARIS					
Can f 1	Lipocalin	Transporter for small hydrophobic molecules, such as lipids and steroid hormones	50–76	Fel d 7	Can f 1 shares 62% aminoacid identity with Fel d 7. The sensitization during childhood has been shown to be predictive marker of dog allergy in adolescence
Can f 2	Lipocalin	Transporter for small hydrophobic molecules, such as lipids and steroid hormones	22–35	Fel d 1	Limited patient-dependent cross-reactivity with Fel d 4
Can f 3	Serum albumin	Regulation of colloid osmotic pressure, transporter for a multitude of metabolites, nutrients, drugs, and other molecules	25–59	Fel d 2, other serum albumins	Serum albumins may play a significant role as cross-reacting allergens in individuals sensitized to dander of multiple animal species
Can f 4	Lipocalin	Transporter for small hydrophobic molecules, such as lipids and steroid hormones	35–59	Equ c 1	Sensitization to Can f 4 has been found in up to 46% of patients allergic to dog.
Can f 5	Kallikrein	Arginine-esterase	71 ^§^	Prostate-specific antigen of human seminal plasma	The Can f 5 amino acid sequence shows no significant similarity to any known animal dander or urinary allergen. Therefore, monosensitization to Can f 5 could be a highly specific marker for allergy to male dogs.
Can f 6	Lipocalin	Transporter for small hydrophobic molecules, such as lipids and steroid hormones	23–61	Fel d 4, Equ c 1	Can f 6 and homologous allergens may contribute to multisensitization and symptoms in individuals allergic to mammals
CAT, FELIS DOMESTICUS					
Fel d 1	Secretoglobin	Its production is related to sexual hormones	60–100	Can f 2	Fel d 1 is the most important allergen in cat allergy, shown to react with IgE from 90% of cat-sensitized individuals, and to account for up to 90% of IgE reactivity to cat dander
Fel d 2	Serum albumin	Regulation of colloid osmotic pressure, transporter for a multitude of metabolites, nutrients, drugs, and other molecules	14–54	Can f 3	Serum albumins may play a significant role as cross-reacting allergens in individuals sensitized to dander of multiple animal species.
Fel d 3 ^#^	Cystatin	Member of the cysteine protease inhibitor family	10		60–90% of cat allergic patients showed IgE binding to rec. Fel d 3 in plaque immunoassay; 10% showed IgE binding in radioallergosorbent test.
Fel d 4	Lipocalin	Transporter for small hydrophobic molecules, such as lipids and steroid hormones	61–63	Can f 6, Equ c 1	Fel d 4 binds IgE at relatively high frequency in cat-sensitive individuals
Fel d 7	Lipocalin	Transporter for small hydrophobic molecules, such as lipids and steroid hormones	38	Can f 1	Fel d 7 has high potential to crossreact with Can f 1, with which shares 62% amino acid identity.
Fel d 8 ^#^	Latherin-like protein		19–20	It has a high degree of homology to horse Equ c 4 and Equ c 5	With an IgE-binding frequency of only 19% among individuals allergic to cat, it is not considered a major cat allergen.

* Sensitization rate in the general population. Sensitization frequencies are variable in different geographic regions. The reported percentage range is the average reported in the literature as extrapolated from the references cited in the Table legend. Allergens are defined as major when are recognized by 50% or more of the sensitized population and minor when are recognized by less than 50% of the sensitized population. ^§^ in Spanish population. ^#^ Not yet available in the commercial IgE immunoassays.

**Table 2:** Comparison between the advantages and disadvantages of the most employed type 2 asthma biomarkers and those of ECP and EDN. AIT, allergen immunotherapy; ECP, eosinophil cationic protein; EDN, eosinophil-derived neurotoxin; FeNO, fractional exhaled nitric oxide; ICS, inhaled corticosteroid; IgE, immunoglobin E; IL, interleukin; mAb, monoclonal antibody. Elaborated from data in [15,56,57,58,60,62,67,68,69,75,76,77,78,79,81,82,83,94].

Biomarker	Usefulness	Advantages	Disadvantages
Blood eosinophil count	It can serve as prognostic biomarker and to predict responsiveness to corticosteroid therapy in asthmatic patients with type 2 inflammation. Baseline value can be used to predict the clinical efficacy of mepolizumab, reslizumab), anti-IL5 receptor antibody (benralizumab) and anti-IL4 receptor antibody (dupilumab).	Easy to realize in the clinical setting, requires minimal patient effort, could be collected across the age spectrum, and it is cost-effective. Circulating blood eosinophil count of ≥300/μL may be helpful to identify a T2 immune biology to initiate therapy with an anti-T2 mAb	The optimal cut-off has yet to be established and its levels may be elevated due to co-existing conditions such as parasitic infestations or decreased due to concomitant medications such as oral corticosteroids. An additional confounding factor is the diurnal variability.
Periostin	The potential use of serum periostin levels is the assessment of greater response to anti-T2-based therapies	The stability of serum periostin over disease progression in adults with asthma (without seasonal effect) and in children between 4 and 11 years of age supports its use as a biomarker for type 2-high asthma.	The presence of several periostin splice variants complicates its detection. The uncertainty regarding its use as potential biomarker in children since baseline periostin levels are higher in children
Serum-specific IgE	The presence of several periostin splice variants complicates its detection. The uncertainty regarding its use as potential biomarker in children since baseline periostin levels are higher in children	The main advantages of specific IgE measurements over the skin prick test are that virtually all available allergens can be tested, and the results are not influenced by antihistamines or eczema. Specific IgE tests are slightly more useful to confirm or reject the suspicion of specific sensitisation to a certain allergen. Assessment of sensitization at the molecular level can play a crucial role before prescribing AIT for the right selection of components.	The interpretation of skin prick tests and specific IgE to whole allergen extracts relies on arbitrary cutoffs, which do not distinguish between pathologic and non-clinically relevant sensitizations.
FeNO	The role of FeNO may be additive as a biomarker in relation to asthma morbidity. In conjunction with peripheral eosinophilia, an elevated FeNO is a risk factor for airway hyperreactivity and uncontrolled asthma.	It can be a valuable tool as it predominantly signifies IL-4 and IL-13 activity. FeNO can be used to adjust ICS dose or as a marker of adherence to treatment. It may be a predictor of response to dupilumab	FeNo may be affected by relevant confounders including smoking, dietary nitrate intake and virus infections.
ECP	In children, ECP may provide complementary value when used together with lung function test and FeNO. Plasma ECP concentrations may be a useful marker of type 2 inflammation in children and may help identify those children at highest risk for recurrent exacerbations who could benefit from corticosteroid treatment.	It can be measured in serum, bronchoalveolar lavage and nasal secretions.	ECP levels are affected by age, smoking, circadian rhythm, and seasonal variation, although only smoking appears to be of clinical significance. Measurement of ECP has shown both time and temperature dependency during serum sampling. There is no internationally established cut-off for serum ECP for use in the diagnosis of asthma.
EDN	It holds great promise in distinguishing wheezing children from children with wheezing triggered by respiratory tract infections, in aiding in the diagnosis of school age childhood asthma as well as to monitor response to montelukast or budesonide in preschool children with asthma. It can be used as biomarker to monitor asthma evolution in adults.	It is easy to obtain from multiple specimen types (e.g., serum, urine, sputum, bronchoalveolar lavage fluid, and nasal lavage fluid) and is not affected by circadian rhythm, smoking or gender differences. Compared to ECP, EDN is significantly less charged, making it easier to work with in routine setting. The quantification of serum EDN is not influenced by the type of storage tube used. It is stable at room temperature or for up to one year when frozen at −20 °C or −80 °C.	Additional studies are needed to validate the benefits of serum EDN for predicting long-term clinical outcomes and selecting right biologics for right patients with severe asthma.

**Table 3:** Wheezing child phenotypes. IgE, immunoglobin E; NA, not available. Elaborated from data in [100,101,102,103,104,105].

Phenotype	Clinical Profile
Transient early wheeze	Onset before the age of 3 years Resolves by the age of 6 years without persistent lung function impairment.
Late-onset wheeze	Onset after 3 years of age and persists in childhood Linked to atopy, reduced lung function and bronchial hyperresponsiveness. Higher likelihood of asthma in adolescence
Persistent wheeze	Starts early life Associated with atopy, high IgE levels, early allergen sensitization and diminished lung function by school age. Higher likelihood of asthma in adolescence
Prolonged early wheeze	Onset between 6 and 54 months of age Not associated with airborne allergen sensitization Weakly associated with higher airway responsiveness and impaired lung function.
Intermediate-onset wheeze	Onset between 18 and 42 months Linked with persisting symptoms, atopy, poor lung function and at more risk of developing asthma in childhood.
Persistent troublesome wheeze	Frequent exacerbations, hospitalizations, and unscheduled visits, reduced lung function, bronchial hyperreactive airways, greater inhalant allergen sensitization in comparison with other phenotypes.

## Data Availability

Not applicable.
